# CNV-GWAS identifies 14 novel Autism-associated loci and distinct effects on Cognitive Ability

**DOI:** 10.21203/rs.3.rs-10933884/v1

**Published:** 2026-10-21

**Authors:** Cecile Poulain, Thomas Renne, Vincent Bourque, Jade England, Florian Benitiere, Audrey Lemaçon, Catherine Proulx, Zohra Saci, Omar Shanta, Worrawat Engchuan, Simon R. Cox, David Porteous, Gail Davies, Paul Redmond, Sarah E. Harris, Gunter Schumann, Zdenka Pausova, Tomas Paus, Celia Greenwood, Laura Almasy, David Glahn, Guillaume Huguet, Sebastien Jacquemont

## Abstract

Copy number variants (CNVs) are well-established risk factors for Autism Spectrum Disorder (ASD). Prior rare-variant association studies have predominantly relied on family-based cohorts, limiting statistical power and identification of novel loci. In addition, it has been challenging to distinguish the effects of CNVs for ASD from the impact on cognitive ability. Here we present a large CNV association study of ASD, analyzing 29,696 autistic and 528,576 control individuals, with cognitive ability data available for over half of the participants. We used two complementary designs, leveraging related and unrelated controls. The unrelated case-control analysis yielded substantially more associations than the family-based design, while effect estimates remained concordant across approaches and showed no evidence of stratification bias. Among recurrent neurodevelopmental CNVs, we identified 19 ASD associations, 8 of which had not been previously linked to ASD, including duplications at 7q11 (ZNF92) and 9p24 (DMRT1), previously associated with schizophrenia. Across the genome, we identified 14 novel candidate CNV loci, several previously associated with risk for schizophrenia, ADHD, or other psychiatric conditions by common and rare variant studies. Cognitive ability data allowed us to delineate two distinct genetic architectures: CNVs predominantly associated with ASD and other mental health conditions, which were enriched for duplications and encompassed less-constrained genes; and CNVs affecting both ASD and cognition, which were enriched for deletions. Together, these findings suggest that classes of variants, rather than genes, may show preferential phenotypic associations.

